# Urachal carcinoma presenting with chronic mucusuria: a case report

**DOI:** 10.1186/1757-1626-1-288

**Published:** 2008-10-30

**Authors:** Ioannis Efthimiou, Charalampos Mamoulakis, Savas Kazoulis, Sarantos Xirakis, Vernadakis Spiros, Ioannis Christoulakis

**Affiliations:** 1 General Hospital of Chania "Agios Georgios" Chania, Crete, TK 73100, Greece

## Abstract

Urachal adenocarcinoma is a rare tumor and represents 0.17–0.34% of all bladder tumors. It has an insidious course and variable clinical presentation. We present a case report of a 58 year old white male with an urachal cyst who suffered irritative voiding symptoms and long term mucusuria, since childhood. After surgical removal of the cyst with a partial cystectomy a mucus adenocarcinoma was diagnosed histologically.

The patient after a negative for metastatic disease screen underwent a completion radical cystectomy with pelvic lymph node clearance. Clinicians should have a high degree of suspicion for these rare tumors.

## Background

Adenocarcinoma of the urachus arises from the urachal remnant. It is a rare and devastating disease, representing 0.17–0.34% of all bladder tumors [1,2]. It is believed that arises from malignant transformation of columnar or glandular metaplastic epithelium. Clinically the distinction of urachal carcinoma from other bladder adenocarcinomas may be difficult especially if the tumor is locally extensive. Histological types include mucinous, enteric, unspecified, signet ring-cell, and mixed variants [3]. In this case report we present a 58 year old white male with a long lasting history of mucusuria, recurrent bacteriuria and lower urinary tract symptoms which finally revealed urachal adenocarcinoma. Also a short review of the literature is presented.

## Case report

**Figure 1:**
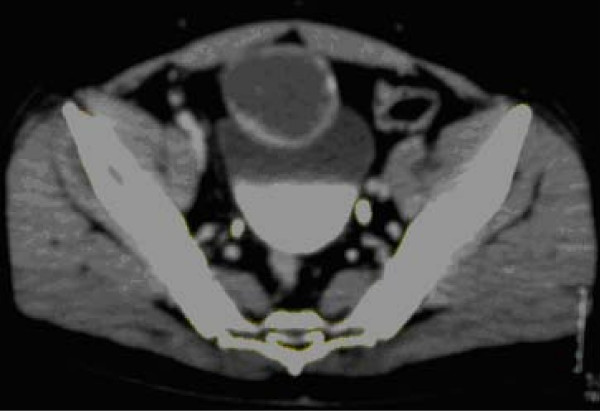
Contrast-enhanced CT scan at level of iliac crests shows a low attenuation cyst overlying the anterosuperior portion of bladder with focal calcifications.

**Figure 2:**
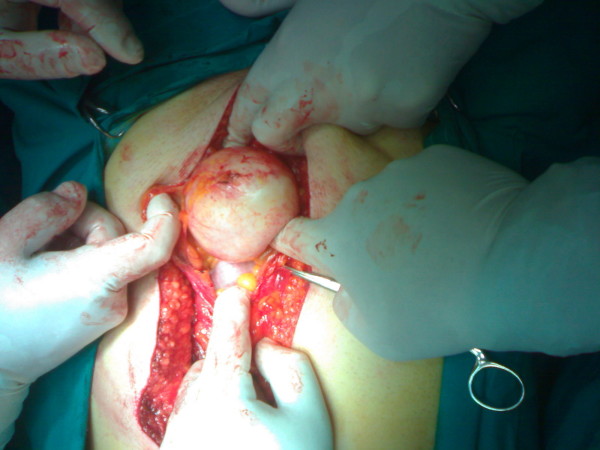
Intraoperative findings of cystic lesion at the dome of the bladder.

A 58 year old white male presented in our department with mucusuria, recurrent bacteriuria and lower urinary tract symptoms. He had been treated in the past for recurrent episodes of prostatitis. He also reported mucusuria that had been present since childhood and for that reason thought it was not unusual. He did not report any hematuria. Abdominal examination revealed a round, smooth, firm, non-tender, suprapubic mass. The prostate gland was soft, painless and of normal size. Biochemical and haematological analysis was normal. An abdominal computerised tomography (CT scan) revealed a 7 × 6 cm round supravesical, midline cyst, with extension into the bladder (figure 1). Antegrade cystography did not reveal any communication between the bladder and the cyst. Rigid cystoscopy under general anaesthesia showed a round and smooth protrusion at the level of the dome and presence of an orifice. The cyst was filled with mucous and there were multiple papillary lesions on the inner surface of the cyst that was biopsied. Histology revealed a urachal adenoma, with no signs of malignancy. The patient underwent an open transperitoneal removal of the cyst (figure 2) and partial cystectomy. Histological examination of the specimen revealed an invasive mucus adenocarcinoma with positive surgical margins. The patient was further evaluated with a pulmonary CT and bone scan which were both negative for metastatic disease. Subsequently he underwent a radical cystectomy and pelvic lymph node dissection with orthotopic neobladder construction.

## Discussion

Most cases of urachal adenocarcinomas occur in the fifth and sixth decade of life. Clinical presentation varies. Hematuria is encountered in more that two thirds of cases. Less common symptoms are mucusuria, lower urinary tract symptoms and a palpable suprapubic mass [4,5]. Mucusuria presents in a 25% of the cases and merits special attention as it may be overlooked for an extremely long time before the correct diagnosis. The most common reason is confusion with lower urinary tract infections such as urethritis and chronic prostatitis.

If after a trial of antibiotic therapy symptoms do not resolve, a full urologic workup with cystoscopy and abdominal ultrasound or CT scan is indicated. CT appearance is of a large mixed solid and cystic lesion with the bulk of the tumor outside the bladder. Extravesical spread is common. In relation to the urinary bladder wall urachal carcinomas may be intramucosal, intramuscular or supravesival, (behind the anterior abdominal wall in the midline) [6]. Tumor calcification is based on mucus production that is encountered in 72%. When present it suggests a urachal adenocarcinoma and it is usually located at the edge of the tumor and it is patchy rather than continuous [2,7].

Treatment consists of open radical or partial cystectomy with pelvic lymph node dissection, and excision of the umbilicus and the urachal ligament [1,2]. Laparoscopic partial cystectomy with lymphadenectomy has been reported as an alternative treatment but long term follow up is required in order to determine the oncologic effect of this treatment [8]. In a recent study from Mayo clinic, staging with TNM system was the main predictor of outcome after surgery for urachal adenocarcinoma [9]. Also negative margin status has been identified as an important factor for long term-survival and adequate local treatment is of paramount value [9,10]. Prognosis does not appear to be significantly influenced by histology and grade of the tumor. Metastatic disease has a poor response to chemotherapy but some tumors may respond to cisplatin based regimens. Overall survival for all stages is 62 months with a 34% of the patients still alive after 5 years.

## Conclusion

Misdiagnosis of urachal carcinoma is still a reality. Accurate diagnosis necessitates a degree of suspicion and appropriate imaging studies. Appropriate local treatment is the key for improved survival.

## Consent

Written informed consent was obtained from the patient for publication of this case report and accompanying images. A copy of the written consent is available for review by the Editor-in-Chief of this journal.

## Competing interests

The authors declare that they have no competing interests.

## Authors' contributions

EI participated in the operating theatre, was a major contributor in writing the manuscript, and submitted the manuscript and photographs. KS participated in the operating theatre and collected the patient data. MC conducted a literature search, preparation of final manuscript including grammarm and stule. VS participated in the operating theatre and assisted in manuscript writing. XS participated in the operating theatre and collected the references. CI participated in the theatre and revised the manuscript. All authors read and approved the final manuscript.
